# The impact of COVID-19 on all-cause mortality and life expectancy: findings from a population-based cohort in uMkhanyakude district, northern KwaZulu-natal

**DOI:** 10.1186/s12963-026-00520-2

**Published:** 2026-10-16

**Authors:** Lusanda Mazibuko, Kobus Herbst, Kathy Baisley, Chodziwadziwa Whiteson Kabudula, Jean Bashingwa, Beth Tippett Bar, Stephen Olivier, Jacob Busang, Siyabonga Nxumalo, Dickman Gareta, Thobeka Mngomezulu, Mark J. Siedner

**Affiliations:** 1 Africa Health Research Institute, Kwa-Zulu Natal, South Africa; 2 University College London, London, UK; 3 DSI-SAMRC South African Population Research Infrastructure Network (SAPRIN), Durban, South Africa; 4 London School of Hygiene and Tropical Medicine, London, UK; 5 MRC/Wits Rural Public Health and Health Transitions Research Unit (Agincourt), School of Public Health, Faculty of Health Sciences, University of the Witwatersrand, Johannesburg, South Africa; 6 Nyanja Health Research Institute, Salima, Malawi; 7 Institute of Social and Preventive Medicine, University of Bern, Bern, Switzerland; 8 Graduate School for Health Sciences, University of Bern, Bern, Switzerland; 9 Massachusetts General Hospital, Boston, Massachusetts USA

**Keywords:** COVID-19 pandemic, Health and demographic surveillance, Excess mortality, Negative binomial regression, Life expectancy

## Abstract

**Background:**

Despite the comprehensive death reporting system in South Africa, significant gaps persist in our understanding of the pandemic’s impact in rural areas.

**Methods:**

We computed monthly death counts stratified by demographic factors from a population-based demographic surveillance cohort in rural KwaZulu-Natal. We fit seasonally adjusted negative binomial regression models to estimate excess mortality during the COVID-19 pandemic period (5 March 2020 to 31 December 2021) compared to the pre-pandemic period (1 January 2015 to 4 March 2020). We then assessed the relative risk of death by health and sociodemographic factors and considering apotential interaction with the COVID-19 pandemic. Finally, we calculated changes in life expectancy at the age of 15 and conducted a decomposition analysis to identify life expectancy changes by age group.

**Results:**

We observed two major periods of excess mortality during the beta and delta waves of the COVID-19 pandemic, respectively. During the pre-pandemic period, people with HIV (PWH) had slightly higher mortality rates (RR = 1.8; 95%CI 1.3 - 2.4) than their HIV-negative counterparts. This excess risk reduced during the COVID-19 period (HIV RR = 1.5; 95%CI 1.2 - 2.0). Similar patterns were noted for individuals with diabetes (pre-pandemic RR = 1.6; 95%CI 1.2 - 2.2 vs during COVID-19 RR = 1.4; 95%CI 1.1 - 1.9). In contrast, individuals with hypertension experienced a 15% relative increase death rate during the pandemic compared to pre-COVID period (p-interaction = 0.003). Similarly, hypertensive PWH had significantly elevated mortality risks during COVID-19 (RR = 2.18; 95% CI: 1.45–3.26, *p* < 0.001). Throughout the study, women consistently had higher life expectancy at age 15 than men. However, women contributed more to pandemic-related life expectancy reductions, along with specific age and health groups.

**Conclusion:**

We found contrasting impacts of co-morbidities on mortality during the COVID-19 pandemic in rural South Africa, and that reductions in life expectancy at age 15 were greater in women than men. These findings emphasize the critical need to consider specific needs of rural populations, and tailor public health strategies that prioritize vulnerable populations and address health inequities to mitigate the effects of pandemics in low-resource settings.

## Background

The COVID-19 pandemic has been one of the most significant public health crises of the twenty-first century, leading to widespread morbidity, mortality, and socio-economic disruptions. Globally, as of 31 December 2021, there have been over 287 million confirmed cases and more than 5.4 million deaths attributed to the virus [1]. However, multiple studies have indicated that official figures likely underestimate the true burden due to underreporting, limited testing capacity, and excess mortality linked to healthcare system disruptions [2–4]. Beyond direct mortality, the pandemic has also had profound secondary effects, including healthcare system strain, disruptions in routine medical care, and economic downturns that exacerbated existing health disparities [5].

In sub-Saharan Africa, the pandemic unfolded differently than in other regions. By December 2021, the WHO African region had reported approximately 7.1 million COVID-19 infections and 150,000 deaths, accounting for 2.5% of global cases and 2.9% of global deaths [1, 6, 7]. However, a modelling study estimated that actual infections in the region could have exceeded 500 million, with only a small fraction officially recorded [7]. Similarly, the COVID-19 Excess Mortality Collaborators suggested that total deaths may have surpassed 2.1 million, far exceeding reported figures [2]. These discrepancies highlight longstanding gaps in disease surveillance, limited access to testing, and the indirect health consequences of the pandemic. Additionally, sub-Saharan Africa’s high burden of infectious and non-communicable diseases (NCDs) likely shaped COVID-19 mortality patterns, with conditions such as HIV, hypertension, and diabetes emerging as major risk factors [8].

South Africa, which has the highest documented COVID-19 burden on the continent, provides a critical case study for understanding the pandemic’s impact in sub-Saharan Africa. By December 2021, the country had reported over 3.4 million laboratory-confirmed cases and 91,145 deaths [9, 10]. However, these figures are likely underestimates, with approximately 201,000 excess deaths from natural causes estimated in South Africa during the same period in 2021, based on historical trends [11, 12]. The majority of these excess deaths are estimated to have occurred during COVID-19 waves [13], which would make COVID-19 the leading cause of death in the country over that period.

Although South Africa’s death reporting system is among the most robust in Africa and has enabled valid estimation of COVID-19 mortality [14], significant gaps remain in understanding the COVID-19 pandemic’s impact within the country. For example, national death registry data are not linked to underlying medical record data, so risk factors for COVID-19 mortality are not discernible from this source. A study from the largely urban Western Cape Province linked medical records to mortality data and found that HIV infection was strongly associated with COVID-19 mortality [15]. However, in rural areas, death reporting is often incomplete or delayed, limiting the generalizability of national findings to these settings [12]. We sought to address these gaps in the literature by leveraging data from a well-characterized demographic and health surveillance area in rural KwaZulu-Natal, South Africa to 1) estimate the incidence of excess mortality among household members, 2) identify the demographic, socioeconomic, and clinical predictors of excess COVID-19 mortality; and 3) determine the impact of COVID-19 on life expectancy. Our over-arching aim was to elucidate the epidemiology of COVID-19 mortality in a low-resource, understudied population in rural South Africa to help inform future epidemic preparedness in the region.

## Methods

### Study design

We analysed longitudinal data spanning the periods 1 January 2015, to 31 December 2021 from a population-based demographic surveillance cohort [16]. Our study included individuals aged 15 and older who were part of the Africa Health Research Institute (AHRI) surveillance population during this reference period.

### Study area and population

**Fig. 1:**
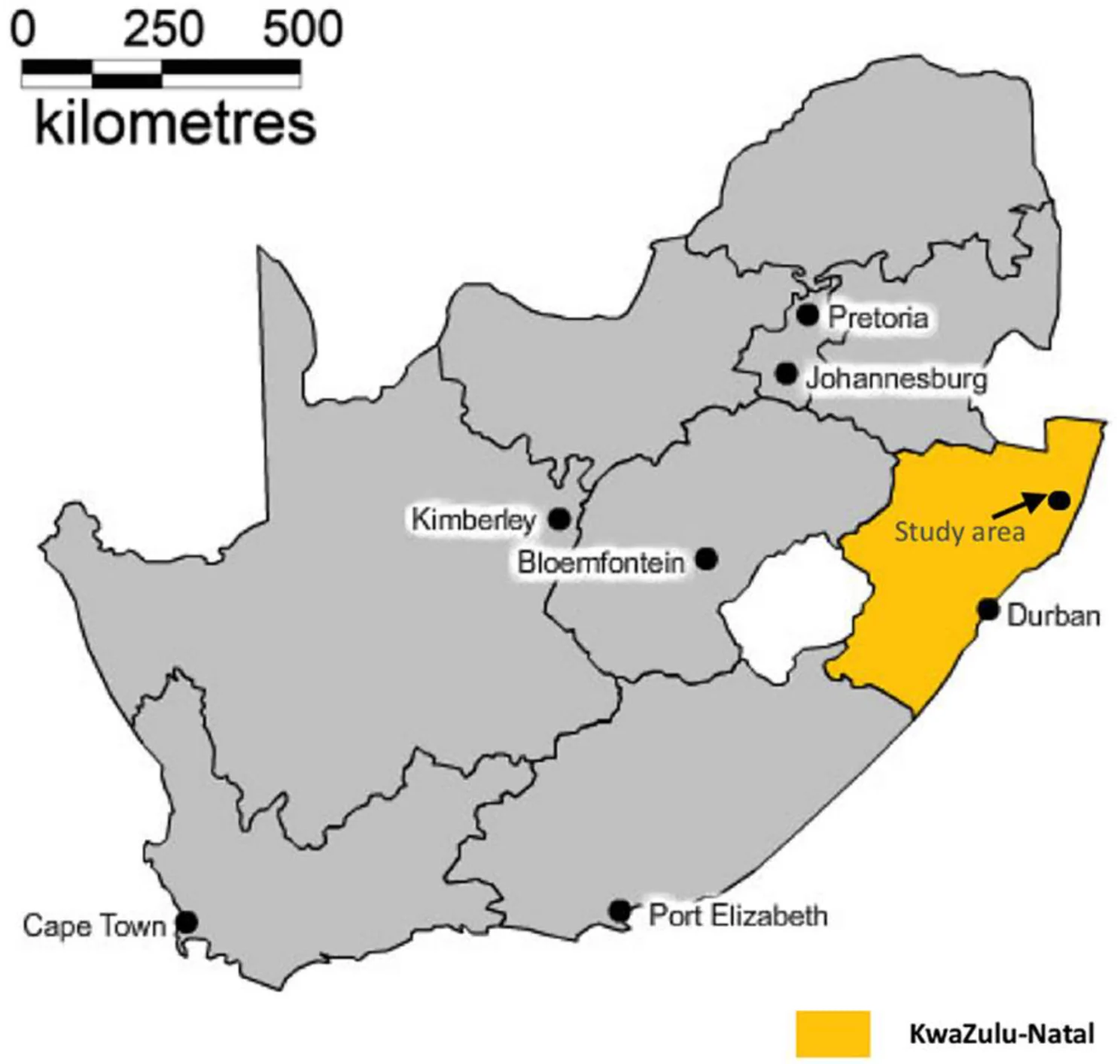
Map showing the AHRI surveillance area within South Africa, adapted from Tlou (2018) [19]

**Fig. 2:**
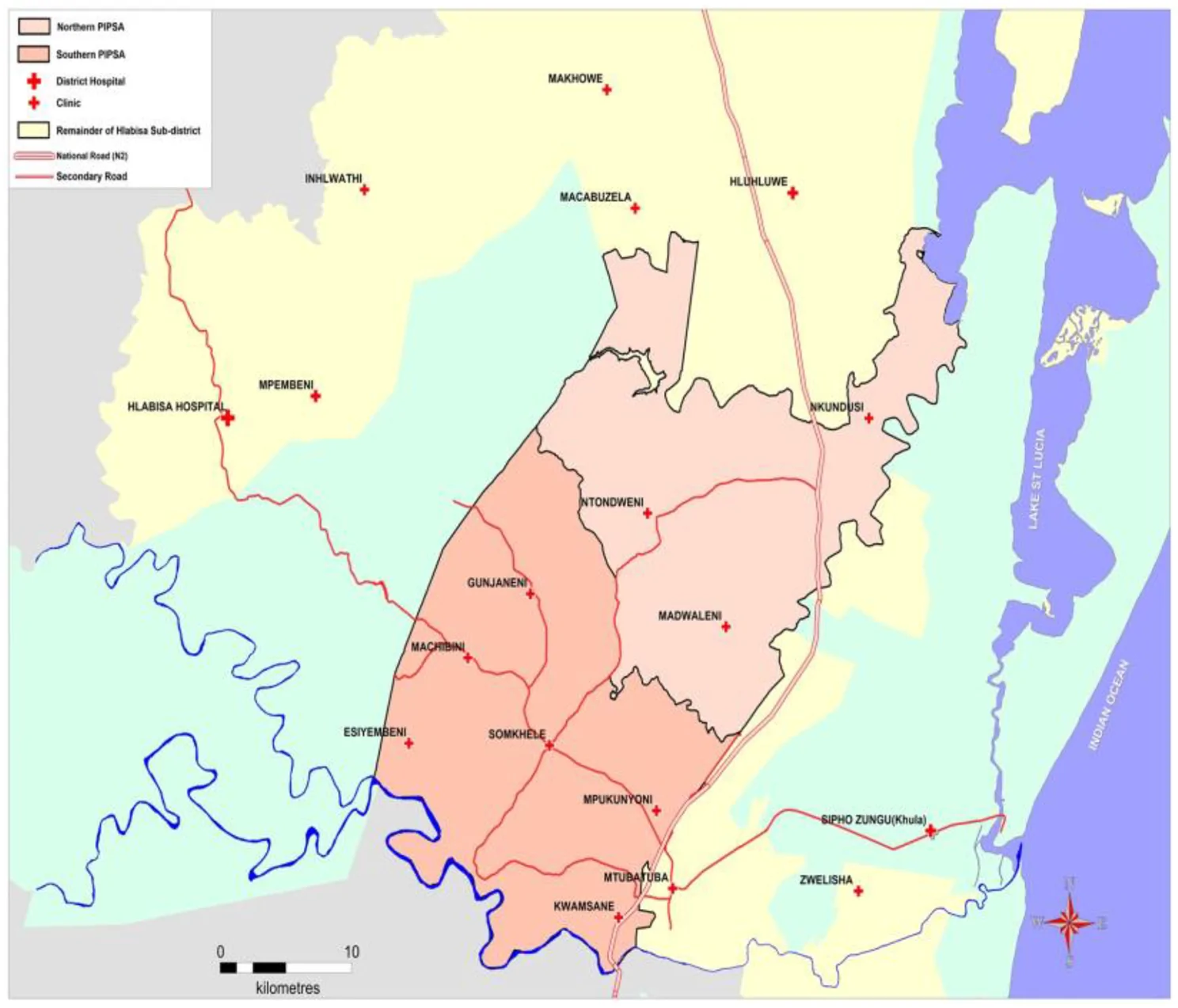
Detailed map of the surveillance area within KwaZulu-Natal, adapted from Gareta et al. (2021) [17]

The AHRI surveillance is situated in the uMkhanyakude District of northern KwaZulu-Natal, South Africa. This region covers approximately 850 square kilometres. It ranks among the areas with lowest socioeconomic status in the country (8). Since its establishment in 2000, AHRI has conducted surveillance across over 30,000 households, which collectively accommodate over 140,000 resident individuals [16, 17]. The surveillance system captures vital data on births, deaths, migrations, and household socio-demographic indicators. Every 15 weeks, all households under surveillance are contacted, forming a vital part of the surveillance cycle. The population in this region faces a significant health challenge, marked by a high prevalence of HIV and non-communicable diseases like hypertension and diabetes [18]. The location of the AHRI surveillance area within South Africa and its geographical extent within northern KwaZulu-Natal are shown in Figs. 1 and 2, respectively.

### Data sources

The AHRI health and demographic surveillance system (HDSS) is one of the most comprehensive population-based surveillance platforms in South Africa. It captures longitudinal data on vital events, including births, deaths, and migrations, as well as socio-demographic and health indicators. Household visits are conducted every 15 weeks to update this information. South African legislation mandates that each death must be registered with the Department of Home Affairs within 72 hours of occurrence [20]. To enhance data accuracy, AHRI employs a multi-step validation process, including verbal autopsy classification for cause-of-death determination and cross-referencing with the national death registry.However, it is important to note that the data is not real-time and relies on the willingness of household respondents to share the information, albeit with a low refusal rate of less than 3%. During the COVID-19 lockdowns, data collection continued, albeit with delays in reporting as the community grappled with the pandemic’s health implications.

To construct the study cohort, we linked mortality data from household surveillance to individual episodes data using pre-existing unique identifiers assigned to participants in the HDSS. Death information collected by routine household surveillance (from which we also extracted socioeconomic status details) was integrated with demographic and health data from clinical screenings. Socioeconomic status (SES) was derived from household surveillance data using principal component analysis (PCA), with the first principal component used as an index of household wealth. This SES index was then categorized into quintiles, with 1 representing the most deprived and 5 the least deprived.

In addition to data collected as part of routine surveillance, we also incorporated health condition data from the Vukuzazi study, a series of roving health fairs conducted between 2018 and 2020 across the surveillance area [18, 21]. This included approximately 18,000 adolescents and adults, representing a subset of the HDSS population, who underwent comprehensive screening for HIV, tuberculosis, diabetes, and hypertension. Health fairs were conducted at designated community locations, and all individuals aged 15 and older were eligible to participate. Objective measurements (i.e. blood pressure measurements, hemoglobin A1c testing, HIV ELISA and sputum for culture and gene Xpert) were collected as part of the Vukuzazi study to establish the diagnosis of each condition [21]. Since all Vukuzazi participants were a subset of the surveillance population, these unique identifiers were already established and consistently maintained across datasets, facilitating direct linkage without data integration challenges. Analyses involving clinical variables were restricted to participants with linked Vukuzazi data for the relevant exposure. Consequently, analytic denominators differed across health conditions according to data availability. Because participation in Vukuzazi was voluntary, these analyses were conducted in a subset of the surveillance population and may therefore be susceptible to selection bias if participants differed systematically from non-participants.

### Definitions

All-cause mortality served as our primary outcome of interest. Our primary exposure of interest was time period, which we divided into pre- and COVID-19 periods. We used the pre-COVID-19 period (1 January 2015 to 4 March 2020) to determine expected mortality, calculated as the number of deaths estimated using a model described below under ‘basic model’. We then estimated excess deaths during the COVID-19 pandemic, characterised as the disparity between expected deaths and the actual number observed during 5 March 2020 to 31 December 2021. Person-time at risk, or exposure time, was determined as the duration from birth until the event of interest occurred or until the end of the study observation period. We censored observation time among participants who remained alive at the conclusion of the study observation period (i.e., 31 December 2021). The population was divided into five socioeconomic strata, with stratum 1 representing the most socioeconomically deprived and 5 representing the least deprived based on a wealth index constructed from a principal component analysis of the list of household assets.

The participants’ health conditions were assessed across several parameters. For diabetes status, individuals were categorized as either having no diabetes or diabetic. The diagnostic tool used involved determining glucose intolerance through the measurement of hemoglobin A1c. Hypertension status was categorized as either no hypertension or hypertensive. Blood pressure levels above 140 mm Hg systolic or 90 mm Hg diastolic were considered hypertensive. The assessment of tuberculosis (TB) included categories of never had TB, and prior TB (based on reported history or suggestive chest X-ray findings) or current TB (confirmed through a positive TB culture or Gene Xpert test). HIV status was determined using ELISA, categorizing individuals as either HIV negative or HIV positive. Lastly, body mass index (BMI) values were categorized into four groups: less than 18.5 kg/m^2^ indicating underweight; 18.5 to 25 kg/m^2^ considered a normal and healthy weight range; 25 to 30 kg/m^2^ categorized as overweight; and greater than 30 kg/m^2^ indicating obesity.

### Statistical analysis

#### Counterfactual construction and estimation of expected mortality

To estimate the number of deaths expected in the absence of the COVID-19 pandemic, we fitted a negative regression model to monthly mortality counts during the pre-pandemic period. Negative binomial regression was selected because preliminary exploration demonstrated overdispersion in the monthly death counts, whereby the variance exceeded the mean. Overdispersion was formally assessed using a likelihood ratio test comparing Poisson and negative binomial models, with evidence favouring the negative binomial model (*p* < 0.05).

The outcome was the monthly number of deaths. An offset equal to the natural logarithm of the monthly population at risk was included to model mortality rates rather than absolute counts. Long-term temporal trends were modelled by including calendar year as a continuous variable. Seasonal variation in mortality was modelled using three pairs of Fourier sine and cosine terms based on calendar month, following established methods for modelling cyclical mortality patterns (14). Age group and sex were included as adjustment variables to account for changes in the demographic composition of the surveillance population over time.

The expected number of deaths in month *t* was modelled as:

$$\begin{aligned}& {\rm{log}}\left\{ {E\left( {{D_t}} \right)} \right\} = {\beta _0} + {\beta _1}{\rm{Yea}}{{\rm{r}}_t} \\ & + \mathop \sum \limits_{i = 2}^I {\beta _i}{\rm{AgeGrou}}{{\rm{p}}_{it}} + {\beta _{I + 1}}{\rm{Se}}{{\rm{x}}_t} \\ & + \mathop \sum \limits_{k = 1}^3 \left[ {{\gamma _k}\sin \left( {{{2\pi kt} \over {12}}} \right) + {\delta _k}\cos \left( {{{2\pi kt} \over {12}}} \right)} \right] + {\rm{log}}\left( {{P_t}} \right)\end{aligned}$$

where

- ${D_t}$ is the observed number of deaths during month *t*;
- $E\left( {{D_t}} \right)$ is the expected number of deaths during month *t*;
- ${\beta _0}$ is the intercept;
- ${\beta _1}$ represents the linear time trend (calendar year);
- ${\beta _i}$ are the regression coefficients for the age-group indicator variables;
- ${\beta _{I + 1}}$ is the regression coefficient for sex;
- ${\gamma _k}$ and ${\delta _k}$ are the coefficients for the *k*th pair of Fourier sine and cosine terms modelling annual seasonality;
- ${P_t}$ is the population at risk during month *t*, included as an offset;
- *t* denotes calendar month.

#### Excess mortality

The counterfactual model was fitted using mortality data from the pre-pandemic period only. Expected deaths during the pandemic period were then predicted from this fitted model while preserving the observed population structure and person-time at risk.

Excess deaths for each month were calculated as the difference between the observed and expected numbers of deaths:

$${\rm{Excess\, death}}{{\rm{s}}_t} = {D_t} - E\left( {{D_t}} \right)$$

Model performance was evaluated internally by assessing predictive performance within the pre-pandemic period using a hold-out validation sample that was not used during model fitting. Calibration was assessed by comparing observed and predicted monthly deaths, while deviance residuals were examined to assess overall model fit and identify systematic departures from model assumptions.

#### Association between demographic and clinical factors and mortality: before and during COVID-19.

Associations between demographic and clinical characteristics and mortality were evaluated among participants aged 15 years and older who participated in the Vukuzazi study. Because Vukuzazi collected health information between 2018 and 2020, clinical characteristics were treated as baseline exposures and were assumed to remain constant during follow-up.

Each demographic or clinical characteristic was examined by extending the basic mortality model to include the exposure of interest, an indicator variable representing the pandemic period (0 = pre-pandemic; 1 = pandemic), and an interaction between the exposure and pandemic period. This interaction tested whether the association between the exposure and mortality differed before and during the COVID-19 pandemic.

The general model took the form

$$\begin{aligned}& {\rm{log}}\left\{ {E\left( {{D_t}} \right)} \right\} = {\beta _0} + {\beta _1}{\rm{Pandemic}} \\ & + {\beta _2}X + {\beta _3}\left( {{\rm{Pandemic}} \times X} \right) + {\beta _4}{\rm{AgeGroup}} \\ & + {\beta _5}{\rm{Sex}} + {\beta _6}{\rm{Year}} + {\rm{Season}} + {\rm{log}}\left( {{P_t}} \right)\end{aligned}$$

where *X* represents the demographic or clinical characteristic under investigation. Relative rates (RRs) and corresponding 95% confidence intervals were estimated by exponentiating the regression coefficients. Age group, sex, calendar year and season were included in all models as prespecified adjustment variables. Statistical significance was assessed using two-sided tests with a significance level of 0.05.

#### Combined health-condition analyses

To investigate the joint effects of multiple health conditions, participants were classified into mutually exclusive categories representing combinations of BMI, hypertension, diabetes, HIV status and tuberculosis. Separate negative binomial regression models were fitted for each grouping using the same modelling framework described above. Models included adjustment for age group, sex, calendar year and season, together with interaction terms between each health-condition grouping and pandemic period to evaluate whether mortality associated with each combination differed between the pre-pandemic and pandemic periods. Relative rates and 95% confidence intervals were estimated for each group relative to the prespecified reference category.

#### Life expectancy

Finally, we sought to determine if and to what extent the COVID-19 pandemic impacted life expectancy in the region. To do so, we estimated life expectancy at age 15 using the period life tables method [22]. We then used the life expectancy decomposition method [23] and divided the population by age group (10-year intervals) and sex or health condition. The segmentation allowed us to identify which age groups, sex or individuals with specific health conditions experienced disproportionate changes in life expectancy during the pandemic. Our analysis used 2019 as the baseline for the pre-pandemic period and 2021 as the second reference year for life expectancy decomposition. The choice of 2021, coinciding with the pandemic’s peak impact on mortality in South Africa, allowed us to accurately estimate how the pandemic, along with its associated challenges and disruptions, influenced overall life expectancy at age 15. Data were imported and analyzed in Stata Version 17 (Statacorp, College Station, TX, USA) and RStudio v 2022.03.

## Results

### Demographic context

Table 1 presents the sociodemographic and health characteristics of cohort participants. The study population consisted of 130,710 individuals aged 15 years or older, who contributed a total of 652,091 person-years at risk from 1 January 2015 to 31 December 2021. During the study period, 6,181 deaths occurred over 652,091 person-years of follow-up, corresponding to an overall mortality rate of 9.5 deaths per 1,000 person-years (95% CI: 9.2–9.7). Mortality rates varied significantly across age categories, with the highest rates observed among individuals aged 65 years and older (mortality rate = 54.0, 95% CI 51.8–56.2 per 1,000 person-years), followed by those aged 45–64 years (14.0, 95% CI 13.3–14.7) and those aged 15–44 years (4.6, 95% CI 4.4–4.8). Mortality rates were slightly higher among men (10.2, 95% CI 9.8–10.5 deaths per 1,000 person-years) compared to women (8.9, 95% CI 8.6–9.2 deaths per 1,000 person-years). We found decreasing cumulative mortality rates with increasing level of socioeconomic status. For example, mortality was 12.6 (95% CI 10.9–14.6) deaths per 1,000 person-years in the most deprived quintile of household wealth and 8.4 (95% CI 8.0–8.9) deaths per 1,000 person-years in the least deprived quintile. With respect to co-morbidities, we found that those with diabetes had the highest mortality rate at 34.2 (95%CI 29.1 - 40.2) per 1000 person-years, followed by those with hypertension at 28.5 (95%CI 25.6 - 31.8) per 1000 person-years and with current/past tuberculosis (26.1, 95% CI 22.6 - 30.1 per 1000 person-years) while those who were HIV positive had a mortality rate of 13.3 (95%CI 12.6 - 14.1) per 1000 person-years. We also observed that those who were at the extreme ends of the BMI categories, i.e. underweight or obese, had elevated mortality rates compared to their counterparts who had a normal BMI. For instance, underweight individuals had a mortality rate of 18.8 (95%CI 13.8 - 25.6) per 1000 person-years while 14 (95%CI 12.2 - 16.0) deaths per 1000 person-years occurred in individuals with obesity.

**Table 1:** Sociodemographic and health characteristics, observed deaths, person-time at risk and mortality rate in the study population, rural uMkhanyakude district, northern KwaZulu-Natal, South Africa, 1 January 2015 – 31 December 2021 (*N* = 130,710)

	Cohort size	Observed deaths	Person-years at risk	Mortality rate per 1000 person years (95% CI)
Study population	130,710	6,181	652,091	9.5 (9.2–9.7)
Age category				
15–44	104,799	2,269	496,041	4.6 (4.4 - 4.8)
45–64	18,816	1,583	112,891	14.0 (13.3 - 14.7)
65+	7,095	2,329	43,160	54.0 (51.8 - 56.2)
Sex				
Male	59,321	3,006	295,767	10.2 (9.8 - 10.5)
Female	71,389	3,175	356,325	8.9 (8.6 - 9.2)
Socioeconomic status				
1 (most deprived)	2,633	180	14,267	12.6 (10.9 - 14.6)
2	9,166	656	57,428	11.4 (10.6 - 12.3)
3	17,063	1,168	110,422	10.6 (10.0 - 11.2)
4	18,493	1,195	119,119	10.0 (9.5 - 10.6)
5 (least deprived)	21,277	1,157	137,295	8.4 (8.0 - 8.9)
Unknown	62,078	1,824	207,009	8.8 (8.4 - 9.2)
HIV status				
Negative	31,380	1,039	125,095	8.3 (7.8 - 8.8)
Positive	16,510	1,141	85,751	13.3 (12.6 - 14.1)
Tuberculosis				
Never had TB	14,884	389	38,183	10.2 (9.2 - 11.2)
TB current/past	2,824	189	7,236	26.1 (22.6 - 30.1)
Diabetes				
No	16,084	435	41,363	10.5 (9.6 - 11.5)
Yes	1,723	148	4,327	34.2 (29.1 - 40.2)
Hypertension				
No	13,247	252	34,179	7.4 (6.5 - 8.3)
Yes	4,583	331	11,594	28.5 (25.6 - 31.8)
BMI category				
Underweight	849	40	2,128	18.8 (13.8 - 25.6)
Normal	6,989	175	17,969	9.7 (8.4 - 11.3)
Overweight	4,012	128	10,300	12.4 (10.4 - 14.8)
Obese	5,846	211	15,072	14.0 (12.2 - 16.0)

### Excess mortality during the COVID-19 pandemic

**Fig. 3:**
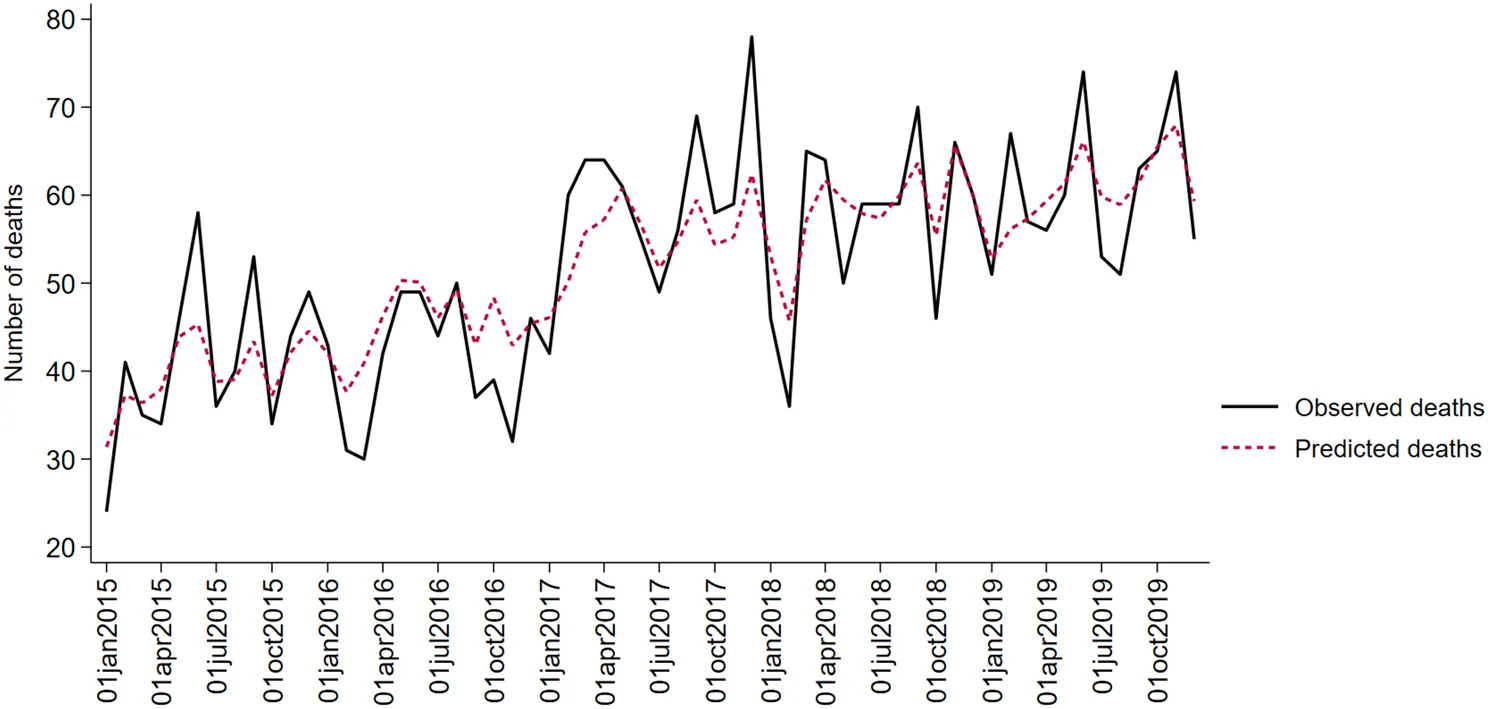
Basic model showing predicted and actual deaths during the pre-pandemic period, 2015–2019

The pre-pandemic expected mortality model fitted the data well with similar predicted and observed deaths (Fig. 3).

**Fig. 4:**
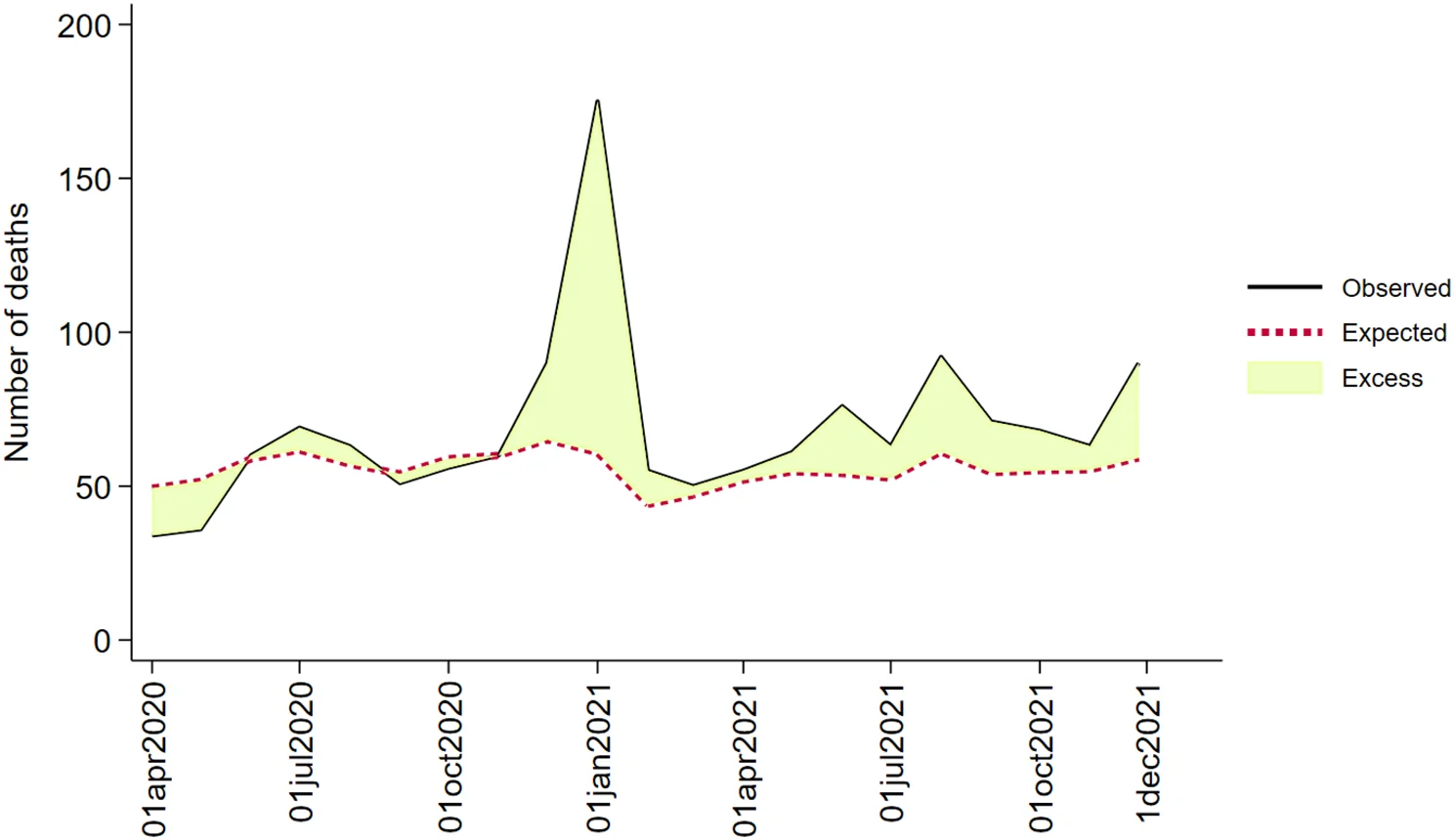
Observed, expected, and excess mortality during the COVID-19 pandemic, rural uMkhanyakude district, northern KwaZulu-Natal, South Africa, 2020 – 2021

We observed two major periods of excess mortality during the COVID-19 pandemic period - from November 2020 to February 2021, which overlapped with the Beta variant wave, and again in July-September of 2021, which overlapped with the Delta wave (Fig. 4). Our analysis ends in December 2021, just as the omicron wave was beginning in the country. While we have limited data on this period, preliminary trends indicate an increase in mortality towards the end of 2021, suggesting that the Omicron wave may have had an impact in this population.

### Association between excess mortality and sociodemographic and health factors during the pandemic

Table 2 presents relative rates (RRs) assessing factors associated with excess mortality pre- and during the COVID-19 pandemic, adjusted for sex, age group, year, and season. In the pre-COVID-19 period, individuals in the socioeconomic status categories 2 to 4 had RRs that indicated no association between their household wealth quintile and mortality, while the highest quintile showed a decreased risk of mortality compared to the lowest quintile. During the pandemic, the second quintile had a reduced RR of death (RR = 0.8; 95%CI 0.5 - 1.2) similar to the highest quintile (RR = 0.7; 95 CI% 0.5 - 1.0). However, these differences between the pre-pandemic and during COVID-19 periods were not statistically significant. Overall, there was no clear trend between SES and mortality in either period, as most SES categories showed no significant association with mortality risk.

**Table 2:** Relative rates (RRs) assessing factors associated with mortality before (May 2018 – 4 March 2020) and during the COVID-19 pandemic (5 March 2020 – 31 December 2021), rural uMkhanyakude district, northern KwaZulu-Natal, South Africa

	Pre-pandemic	During COVID-19	
	**Adjusted relative rate (95% CI)**	**Adjusted relative rate (95% CI)**	**p interaction**
Socioeconomic status			
1 (most deprived)	1 (Reference)	1 (Reference)	
2	1.1 (0.9 - 1.3)	0.8 (0.5 - 1.2)	0.312
3	1.0 (0.8 - 1.2)	1.0 (0.7 - 1.5)	0.865
4	1.0 (0.8 - 1.1)	1.0 (0.7 - 1.4)	0.888
5 (least deprived)	0.8 (0.7 - 1.0)	0.7 (0.5 - 1.0)	0.093
HIV Status			
Negative	1 (Reference)	1 (Reference)	
Positive	1.8 (1.3 - 2.4)	1.5 (1.2 - 2.0)	0.002
Tuberculosis			
Never had TB	1 (Reference)	1 (Reference)	
TB (Current/past)	1.8 (1.3 - 2.4)	1.2 (0.9 - 1.5)	0.248
Diabetes			
No	1 (Reference)	1 (Reference)	
Yes	1.6 (1.2 - 2.2)	1.4 (1.1 - 1.9)	0.009
Hypertension			
No	1 (Reference)	1 (Reference)	
Yes	1.3 (1.0 - 1.8)	1.5 (1.1 - 1.9)	0.003
BMI category			
Normal	1 (Reference)		
Underweight	2.7 (1.7 - 4.5)	1.6 (1.0 - 2.7)	0.071
Overweight	0.9 (0.6 - 1.3)	0.8 (0.6 - 1.2)	0.317
Obese	0.9 (0.7 - 1.3)	0.9 (0.6 - 1.2)	0.400

The above table is from a negative binomial model of monthly counts, taking into account the number at risk in each month as the denominator. The analysis is adjusted for sex, age group, year and season (by including Fourier terms). Each morbidity/health factor is examined separately and included in the model with an interaction with pandemic period

Mortality rates were higher in participants with HIV compared to HIV-negative individuals during the pre-pandemic (RR = 1.8; 95%CI 1.3 - 2.4) and COVID-19 (HIV RR = 1.5, 95%CI 1.2 - 2.0) periods and the interaction was statistically significant p-interaction = 0.002). A similar observation was made regarding participants with TB and diabetes in both periods, except the difference was not statistically significant for TB. Further, hypertensive participants had increased RR during COVID-19 compared to the pre-pandemic period (p interaction = 0.003). Finally, only underweight individuals exhibited higher mortality rates pre-and during the pandemic compared to those with normal BMI. Although during COVID-19 period, the risk was reduced (RR = 1.6 vs RR = 2.7 pre-pandemic), there was weak evidence to support the difference between time periods (p-interaction = 0.071).

Table 3 presents RRs assessing the combined effects of BMI, diabetes, hypertension, TB, and HIV on mortality pre- and during the COVID-19 pandemic, adjusted for sex, age, year, and season. Pre-pandemic, underweight non-diabetic individuals had a significantly higher mortality rates compared to those with normal BMI (RR = 17.79; 95% CI: 4.31–73.41). However, during the pandemic, this risk declined notably (RR = 7.28; 95% CI: 1.01–52.48, p-interaction = 0.049). Among diabetics, those who were underweight experienced higher mortality across periods, but with weaker evidence of temporal differences (*p* = 0.074).

**Table 3:** Relative rates assessing the combined effects of BMI, diabetes, hypertension, TB, and HIV on mortality pre- and during the COVID-19 pandemic, adjusted for sex, age, year, and season

	Pre-pandemic (RR, 95% CI)	During COVID-19 (RR, 95% CI)	p-interaction
**BMI and diabetes**			
Normal weight, Not Diabetic	1 (Reference)	1 (Reference)	
Underweight, not diabetic	17.79 (4.31 - 73.41)	7.28 (1.01 - 52.48)	0.049
Underweight and diabetic	2.68 (1.60 - 4.48)	1.62 (0.96 - 2.73)	0.074
Normal weight, Diabetic	1.82 (0.89 - 3.72)	1.80 (0.99 - 3.26)	0.054
Overweight/Obese, Diabetic	1.32 (0.88 - 1.98)	1.23 (0.86 - 1.74)	0.255
Overweight/Obese, Not Diabetic	0.81 (0.58 - 1.15)	0.82 (0.61 - 1.10)	0.177
**BMI and hypertension**			
Normal weight, not hypertensive	1 (Reference)	1 (Reference)	
Underweight and hypertensive	2.62 (1.10 - 6.26)	2.19 (0.93 - 5.15)	0.073
Underweight, not hypertensive	2.84 (1.58 - 5.11)	1.63 (0.86 - 3.06)	0.131
Normal weight, hypertensive	1.07 (0.63 - 1.80)	1.41 (0.92 - 2.18)	0.117
Overweight/Obese and hypertensive	1.09 (0.72 - 1.64)	1.21 (0.84 - 1.73)	0.31
Overweight/Obese, not hypertensive	0.69 (0.44 - 1.07)	0.74 (0.50 - 1.08)	0.123
**Hypertension and diabetes**			
Neither hypertensive nor diabetic	1 (Reference)	1 (Reference)	-
Non-hypertensive, diabetic	1.27 (0.68 - 2.36)	1.57 (0.95 - 2.60)	0.08
Hypertensive, not diabetic	1.13 (0.80 - 1.58)	1.51 (1.13 - 2.02)	0.005
Hypertensive and diabetic	1.99 (1.36 - 2.92)	1.91 (1.35 - 2.72)	<0.001
**TB and HIV**			
Never had TB and HIV-negative	1 (Reference)	1 (Reference)	
Never had TB and HIV-positive	1.44 (1.01 - 2.05)	1.50 (1.12 - 2.00)	0.006
Have (had) TB and HIV-negative	1.04 (0.60 - 1.80)	1.31 (0.85 - 2.01)	0.222
Have (had) TB and HIV-positive	2.26 (1.51 - 3.39)	1.33 (0.87 - 2.02)	0.189
**HIV and hypertension**			
HIV-negative and not hypertensive	1 (Reference)	1 (Reference)	
HIV-negative, hypertensive	1.94 (1.33 - 2.81)	1.64 (1.19 - 2.25)	0.002
HIV-positive, not hypertensive	2.62 (1.75 - 3.92)	1.64 (1.15 - 2.34)	0.007
HIV-positive and hypertensive	1.88 (1.10 - 3.20)	2.18 (1.45 - 3.26)	<0.001
**HIV and diabetes**			
HIV-negative and not diabetic	1 (Reference)	1 (Reference)	-
HIV-positive and diabetic	2.30 (1.18 - 4.48)	2.19 (1.29 - 3.73)	0.004
HIV-positive, not diabetic	1.94 (1.39 - 2.71)	1.45 (1.09 - 1.94)	0.011
HIV-negative, diabetic	1.97 (1.37 - 2.82)	1.51 (1.10 - 2.08)	0.011
**HIV and BMI**			
HIV-negative and normal weight	1 (Reference)	1 (Reference)	
HIV-positive and normal weight	2.43 (1.48 - 4.00)	1.75 (1.14 - 2.66)	0.01
HIV-negative and underweight	2.52 (1.23 - 5.14)	1.42 (0.69 - 2.90)	0.339
HIV-positive and underweight	7.09 (3.62 - 13.89)	3.13 (1.53 - 6.43)	0.002
HIV-negative and overweight/obese	1.18 (0.73 - 1.90)	1.12 (0.76 - 1.65)	0.582
HIV-positive and overweight/obese	1.29 (0.67 - 2.49)	0.78 (0.43 - 1.44)	0.432
**TB and BMI**			
Never had TB and normal weight	1 (Reference)	1 (Reference)	-
Never had TB and underweight	2.13 (1.11 - 4.09)	1.19 (0.59 - 2.37)	0.631
Never had TB and Overweight/obese	0.87 (0.62 - 1.23)	0.89 (0.67 - 1.18)	0.409
Have (had) TB and underweight	4.44 (2.30 - 8.59)	2.79 (1.39 - 5.61)	0.004
Have (had) TB and normal weight	1.20 (0.68 - 2.12)	1.19 (0.74 - 1.91)	0.47
Have (had) TB + Overweight/obese	1.15 (0.67 - 1.98)	0.85 (0.51 - 1.41)	0.524
**TB and diabetes**			
Never had TB and not diabetic	1 (Reference)	1 (Reference)	
Never had TB and diabetic	1.66 (1.18 - 2.33)	1.47 (1.09 - 1.96)	0.011
Have (had) TB, and not diabetic	1.51 (1.03 - 2.22)	1.19 (0.84 - 1.68)	0.326
Have (had) TB and diabetic	2.74 (1.47 - 5.13)	1.79 (0.94 - 3.4)	0.077
**TB and hypertension**			
Never had TB and not hypertensive	1 (Reference)	1 (Reference)	
Never had TB, hypertensive	1.54 (1.12 - 2.13)	1.59 (1.2 - 2.1)	0.001
Have (had) TB, not hypertensive	2.06 (1.33 - 3.19)	1.38 (0.9 - 2.11)	0.145
Have (had) TB and hypertensive	1.63 (0.95 - 2.81)	1.69 (1.07 - 2.68)	0.025

Participants with either hypertension or diabetes had increased mortality rates during the pandemic. For diabetic participants, there was marginal evidence of a difference in mortality risks between time periods (p-interaction = 0.080). However, individuals with hypertension alone experienced a significant increase in mortality risks during the pandemic compared to pre-pandemic levels (p-interaction = 0.005). Hypertensive diabetics had the highest RRs pre-pandemic (RR = 1.99; 95% CI: 1.36–2.92), with a slight decrease in RR during the pandemic (RR = 1.91; 95% CI: 1.35–2.72, *p* < 0.001).

TB and HIV showed distinct patterns. HIV-positive individuals with a history of TB had reduced mortality rates during COVID-19 (RR = 1.33) compared to pre-pandemic (RR = 2.26), though this difference was not statistically significant (*p* = 0.189). However, participants who were living with HIV and had no history of TB experienced a 4.2% increase in mortality rate during the pandemic, with strong evidence of temporal differences (p-interaction = 0.006).

For HIV and BMI, all relative risks decreased during the pandemic to varying degrees. However, evidence of interaction was only observed for HIV-positive participants with normal weight, while other BMI categories showed no significant temporal differences. Similarly, for TB and BMI, most estimates showed reductions during the pandemic without evidence of interaction. Exceptions were observed for diabetes: participants with no history of TB and diabetes exhibited significant temporal differences (p-interaction < 0.001), while those with a history of TB and diabetes had marginal evidence of interaction (p-interaction = 0.077). Several combined comorbidity analyses yielded wide confidence intervals, reflecting sparse observations within some exposure combinations. These subgroup findings should therefore be interpreted cautiously and considered exploratory.

For hypertensive HIV-positive participants, mortality risks increased further during COVID-19 (RR = 2.18; 95% CI: 1.45–3.26), with a statistically significant interaction (*p* < 0.001). Similarly, TB patients who were underweight had elevated mortality risks in both periods, though the risk was significantly lower during the pandemic (*p* = 0.004).

### Life expectancy at age 15 reduction during the pandemic

**Fig. 5:**
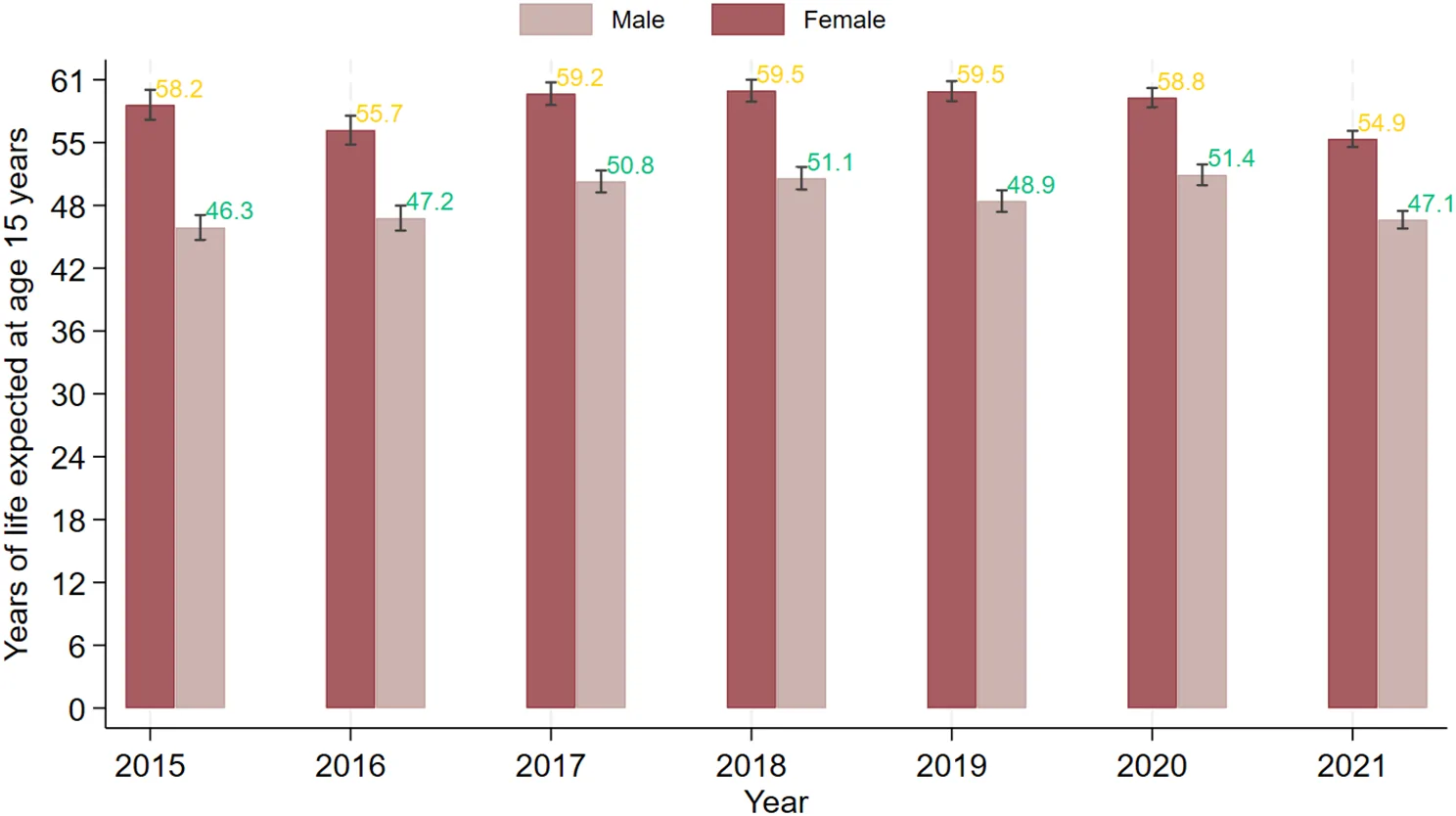
Life expectancy at age 15 years, rural uMkhanyakude district, northern KwaZulu-Natal, South Africa, 2015 – 2021

During the entire observation period, women had a higher life expectancy at age 15 years than men. In the beginning of the study period, women had a life expectancy of 70.1 years (95%CI 68.1 - 72.1) at age 15 years compared to their male counterparts who had a life expectancy of 61.2 years (95%CI 59.3–63.0, Fig. 5). From 2015 to 2020, life expectancy remained fairly stable in women and slightly increased in men up to 65.6 (95%CI 64.1–67.1). However, we detected a significant drop in life expectancy from 2020 to 2021, in both sexes, with a decrease in LE of 3.3 years in women and 3.2 years in men in that period.

**Fig. 6:**
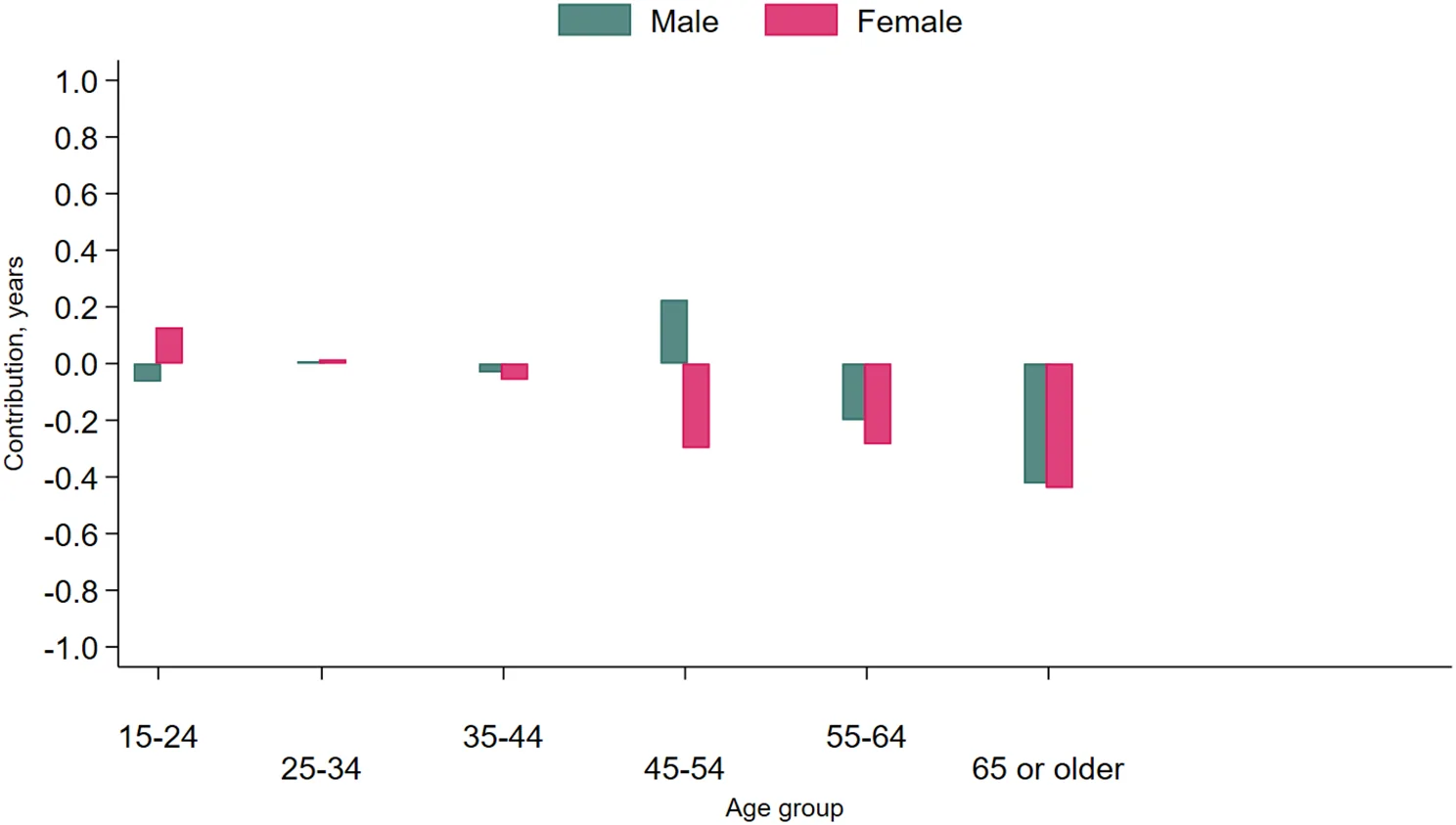
Age contribution to life expectancy at age 15 years changes by sex: comparing pre- and during COVID-19 period, KwaZulu-Natal, South Africa, 2019 and 2021

When we considered decomposed life expectancy at age 15 years, we noted that adolescent boys and young men (15–24) and men aged 45–54 contributed to reduction in life expectancy in this population. For both sexes, ages 35–44 years and 55 years and older contributed most years to losses in life expectancy. Despite this, women generally had a higher contribution to life expectancy reductions than men (Fig. 6).

**Fig. 7:**
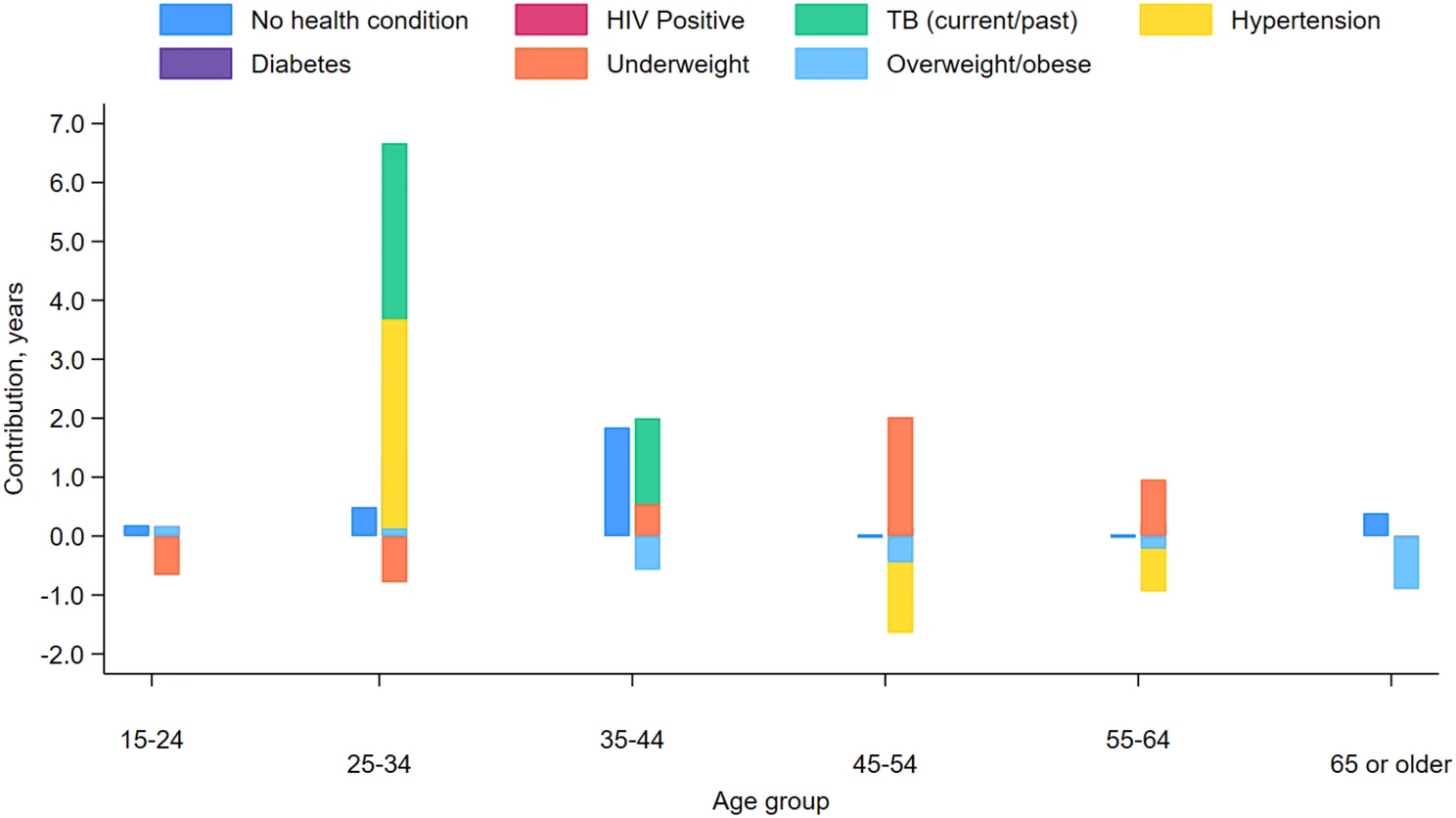
Life expectancy at age 15 changes by age group and health condition status: comparing the pre- and during the pandemic period, rural uMkhanyakude district, northern KwaZulu-Natal, South Africa, 2019 and 2021

Segregating by health conditions and age groups (Fig. 7), we noted that 15–34-year-old underweight individuals, overweight individuals of any age group, and 45–64-year-old people with hypertension contributed most years to the loss in life expectancy years.

## Discussion

In this population-based cohort study from rural KwaZulu-Natal, we observed substantial excess mortality during the Beta and Delta epidemic waves, accompanied by marked reductions in life expectancy, particularly among adults aged 55 years and older. Mortality remained higher among individuals living with HIV, diabetes and hypertension throughout the study period. However, while the relative excess associated with HIV and diabetes was attenuated during the pandemic, the association with hypertension became stronger. Collectively, these findings demonstrate that considerable pandemic-period excess mortality occurred within this rural population, despite early suggestions that lower population density might protect rural communities. Although the mechanisms underlying these findings cannot be established directly from our data, the high burden of chronic infectious and non-communicable diseases in this setting, together with documented disruptions to healthcare services during the pandemic, are plausible contributing factors supported by previous studies [15, 24–26].

The impact of COVID-19 on mortality in KwaZulu-Natal was profound, with the South African Medical Research Council estimating 58,627 excess deaths between 3 May 2020 and 1 January 2022 [11]. Our findings demonstrate that these province-wide mortality patterns were also evident at the district level within a rural population. Mortality during the pandemic was approximately 60% higher than expected based on pre-pandemic trends, with two pronounced periods of excess mortality corresponding to the Beta and Delta epidemic waves. The close agreement between the timing of these mortality peaks and provincial epidemic trends provides reassurance that our counterfactual model adequately captured expected mortality and that the observed excess deaths reflected the broader progression of the pandemic in KwaZulu-Natal. Importantly, these findings demonstrate that substantial excess mortality was not confined to South Africa’s urban centres but also occurred within predominantly rural communities characterised by a high burden of HIV and non-communicable diseases. Although our study was not designed to identify the mechanisms underlying these mortality patterns, several contextual factors may have contributed. Rural KwaZulu-Natal has one of the highest HIV prevalences globally alongside a growing burden of hypertension and diabetes [18], conditions consistently associated with poorer COVID-19 outcomes [15, 24]. Furthermore, disruptions to routine healthcare services and reduced access to acute care during successive epidemic waves may have contributed to both direct and indirect mortality [26]. Social and cultural factors may also have influenced transmission dynamics. Qualitative work from rural Eastern Cape suggests that funeral practices, which often involve extensive community participation, facilitated increased social contact during the pandemic [27]. Although we were unable to evaluate these mechanisms directly, similar social structures may have influenced transmission within our study setting. Most South African studies of excess mortality have relied on national surveillance systems or have focused on urban populations [11, 12, 28]. By leveraging longitudinal data from a well-characterised rural demographic surveillance system, our study provides important population-based evidence that the mortality consequences of the COVID-19 pandemic extended beyond urban settings and highlights the need for preparedness strategies that explicitly address the unique vulnerabilities of rural populations.

The associations between HIV, diabetes, and hypertension and mortality observed in our study are broadly consistent with findings from national and provincial studies in South Africa, which have consistently identified these conditions as important determinants of mortality during the COVID-19 pandemic. These studies suggest that the pandemic did not create entirely new patterns of vulnerability but rather amplified existing inequalities in populations already burdened by chronic infectious and non-communicable diseases. Our findings extend this evidence by demonstrating that these relationships were also evident within a rural population, where the coexistence of HIV and an increasing burden of non-communicable diseases presents a unique epidemiological context. Interestingly, although mortality remained elevated among individuals living with HIV and diabetes throughout the study period, the relative excess associated with these conditions was attenuated during the pandemic compared with the pre-pandemic period, whereas the association with hypertension became more pronounced. One possible explanation is that mortality increased across the broader population during successive COVID-19 waves, thereby reducing the relative differences between individuals with and without some chronic conditions. Alternatively, changes in access to HIV services, chronic disease management, or healthcare-seeking behaviour during the pandemic may also have influenced these patterns [29]. Although our analyses cannot distinguish between these mechanisms, the persistence of elevated mortality across all three conditions reinforces the importance of maintaining continuity of care for individuals living with chronic diseases during future epidemics.

Our combined analyses provide further insight into the cumulative impact of multimorbidity during the pandemic. Individuals living with both hypertension and either diabetes or HIV consistently experienced the highest mortality, supporting growing evidence that vulnerability to severe infectious disease is shaped not only by individual conditions but also by the accumulation of chronic disease [24, 30]. This finding is particularly relevant in South Africa, where multimorbidity is becoming increasingly common as populations age while HIV remains highly prevalent [18, 24]. Rather than acting independently, these conditions likely reflect overlapping biological vulnerability together with greater healthcare needs, making individuals more susceptible to adverse outcomes during periods of health system strain. At the same time, several combined subgroup estimates were accompanied by wide confidence intervals, reflecting relatively small numbers of events. These estimates should therefore be interpreted cautiously and regarded primarily as hypothesis-generating. For example, although underweight individuals without diabetes appeared to experience lower relative mortality during the pandemic than before the pandemic, the substantial statistical uncertainty surrounding this estimate precludes strong conclusions and warrants confirmation in larger studies.

The tuberculosis findings warrant particularly careful interpretation. Previous studies have consistently identified active or previous tuberculosis as an important predictor of severe COVID-19 outcomes and mortality, especially in settings with high HIV prevalence [15, 24]. In contrast, we observed lower relative mortality associated with tuberculosis during the pandemic than during the pre-pandemic period. Several explanations may account for this apparent discrepancy. First, our tuberculosis exposure combined both current and previous tuberculosis, resulting in a heterogeneous group whose underlying risk may differ from cohorts restricted to active disease. Second, individuals with tuberculosis are often closely engaged with healthcare services through structured treatment and follow-up programmes, particularly in high HIV-burden settings, which may have facilitated earlier diagnosis and management of acute illness during the pandemic [24, 25]. It is also possible that the observed associations reflect differences in the underlying population at risk, including survivor bias or residual confounding, rather than a true reduction in mortality risk. However, confidence intervals surrounding several tuberculosis-related estimates were wide, reflecting limited precision within subgroup analyses, and chance cannot be excluded as an explanation for these findings. Consequently, these results should be interpreted cautiously and confirmed in larger longitudinal studies with more detailed characterisation of tuberculosis disease status and treatment.

The reductions in life expectancy observed between 2020 and 2021 demonstrate that the pandemic temporarily reversed recent gains in survival within this rural population. The declines of 3.3 years among women and 3.2 years among men are consistent with national and international evidence showing that the COVID-19 pandemic substantially reduced life expectancy across diverse populations [30, 31]. Our findings extend this evidence to a rural South African setting, where evidence has been comparatively limited. Importantly, they suggest that the mortality consequences of the pandemic were sufficiently severe to offset several years of improvements in population survival within a relatively short period. Similar patterns have been reported across KwaZulu-Natal and internationally, with the 2021 Global Burden of Disease Study identifying KwaZulu-Natal as one of the regions experiencing the largest declines in life expectancy worldwide [30]. Nationally, life expectancy at age 60 declined by 1.3 years among men and 1.6 years among women [31]. Collectively, these findings indicate that the profound mortality impact observed nationally and internationally was similarly experienced within this rural community.

Although women maintained a longer life expectancy than men throughout the study period, they experienced slightly greater reductions in life expectancy during the pandemic. This finding suggests that the well-established female survival advantage narrowed during a period of substantial excess mortality, although it was not eliminated [32, 33]. Several explanations may account for this pattern. Women often undertake greater domestic and caregiving responsibilities, potentially increasing opportunities for exposure during infectious disease outbreaks [34–36]. In addition, women constitute a substantial proportion of South Africa’s frontline healthcare workforce, placing many at increased occupational risk during successive COVID-19 waves [35, 37]. Differences in healthcare utilisation, occupational exposure and caregiving roles may therefore have contributed to the greater reductions observed among women. However, these explanations should be interpreted as plausible hypotheses rather than causal inferences.

The age-specific decomposition of life expectancy losses provides additional insight into how the mortality burden of the pandemic was distributed across the population. Among men, the greatest contributions to life expectancy loss arose from younger adults aged 15–24 years and those aged 45–54 years, whereas among women the largest contributions were observed among those aged 35–44 years and 55 years and older. These findings indicate that the mortality impact of the pandemic was not confined to the oldest age groups but affected multiple stages of the life course. The greater contribution of younger adult men is consistent with studies suggesting that differences in occupational exposure, mobility and social contact patterns may have influenced infection risk during the pandemic [38, 39]. Conversely, the larger contribution of older women to life expectancy losses may reflect the combined effects of age-related vulnerability and the greater reductions in life expectancy observed among women overall. Although our study was not designed to investigate the mechanisms underlying these age- and sex-specific patterns, the findings highlight that the population-level consequences of the pandemic were distributed unevenly across demographic groups.

The differential contributions of hypertension and abnormal weight to reductions in life expectancy further emphasise the importance of chronic disease in shaping population resilience during public health emergencies. These findings are consistent with previous work from this surveillance population demonstrating substantial unmet need for the diagnosis and management of hypertension and abnormal weight, with approximately 53 and 78% of affected individuals, respectively, reporting unmet healthcare needs [40]. Poor detection and suboptimal long-term management of chronic conditions may increase susceptibility to severe illness during periods of health system disruption, thereby amplifying their contribution to premature mortality. Although our analyses cannot establish whether unmet healthcare needs directly mediated the observed reductions in life expectancy, they suggest that strengthening routine prevention, early diagnosis and long-term management of chronic diseases should remain a central component of pandemic preparedness, particularly in rural settings with a high burden of multimorbidity.

Lastly, the observed effect of overweight/obesity on life expectancy during the pandemic for those aged between 15 and 34 years warrants discussion for two reasons. First, the relationship between health conditions and life expectancy is heterogeneous, as these results highlight, and public health interventions should take age and particular health conditions into account. These age groups represent a segment of the population that is often overlooked in the discussion of non-communicable diseases (NCDs) and their associated risks [41–43]. Although NCDs are usually associated with older age groups, it is important to note that younger people especially those living with HIV can also suffer, sometimes from undiagnosed or untreated conditions [42, 44, 45]. This is a matter of interest in our study population as there is an established high incidence of HIV among its young people along with evidence of concurrent NCDs and other infectious diseases [18, 46]. Second, the findings highlight a potential deficit in health care access and NCD awareness among younger adults in this setting. The contribution of overweight and obesity among younger adults to reductions in life expectancy is noteworthy because non-communicable diseases are often perceived as conditions affecting older populations. Our findings suggest that younger adults living with obesity may also represent an important vulnerable group in rural South Africa, reinforcing the need for earlier identification and management of chronic disease risk factors.

## Limitations

This study has several limitations. First, although excess mortality provides a robust measure of the overall mortality impact of a public health emergency, it relies on estimating the number of deaths expected in the absence of the pandemic rather than directly measuring COVID-19-attributable mortality. The close temporal correspondence between excess mortality and successive COVID-19 epidemic waves, observed both globally and in South Africa, suggests that a substantial proportion of the excess deaths recorded during this period were likely attributable, either directly or indirectly, to the COVID-19 pandemic [1, 2, 47]. However, the absence of cause-specific mortality data precludes direct attribution of excess deaths to COVID-19 and prevents differentiation between deaths caused by SARS-CoV-2 infection and those resulting from indirect consequences of the pandemic, such as disruptions to healthcare services. Furthermore, although South Africa’s vital registration system is well established, under-registration of deaths and incomplete ascertainment of COVID-19 deaths during the pandemic remain important considerations, particularly in rural settings. Bradshaw et al. (2022) estimated that reported COVID-19 mortality substantially underestimated the true burden, with excess mortality suggesting that actual COVID-19 deaths may have been 2.5 to 3 times higher than officially reported [48]. Second, although life expectancy changes effectively summarise mortality impacts, they are influenced by the population structure and may not reflect all underlying health dynamics during the pandemic. Third, the health condition data from the Vukuzazi survey (2018–2020) have inherent limitations. The shorter exposure window restricts a comprehensive assessment of long-term health outcomes. Additionally, analyses involving health conditions were restricted to participants who attended the Vukuzazi health screening programme and had complete data for the relevant variables. Because participation was voluntary, this subset may not be fully representative of the underlying surveillance population, introducing the potential for selection bias. Furthermore, health conditions were measured once between 2018 and 2020 and treated as fixed over time. Consequently, changes in BMI, HIV status, diabetes and hypertension occurring during the pandemic were not captured, potentially resulting in exposure misclassification. These limitations should be considered when interpreting the findings but are unlikely to alter the overall conclusion that the COVID-19 pandemic was associated with substantial excess mortality and reductions in life expectancy within this rural population.

## Conclusion

This study demonstrates that the COVID-19 pandemic was associated with substantial excess mortality and marked reductions in life expectancy within a rural South African population. The observed mortality burden likely reflected both direct and indirect consequences of the pandemic, disproportionately affecting individuals living with HIV, hypertension and diabetes, and resulting in important losses in life expectancy across multiple demographic groups. Together, these findings highlight how pre-existing burdens of chronic infectious and non-communicable diseases amplified the population-level consequences of the pandemic in a rural, resource-constrained setting.

By leveraging longitudinal data from a well-characterised demographic surveillance system, our study provides important population-based evidence that the mortality consequences of the COVID-19 pandemic extended beyond South Africa’s urban centres. These findings underscore the importance of strengthening chronic disease prevention and management, maintaining continuity of essential healthcare services during public health emergencies, and ensuring that pandemic preparedness strategies explicitly address the unique vulnerabilities of rural populations with a high burden of multimorbidity.

## Data Availability

The datasets generated and/or analyzed during the current study are available on GitHub at the persistent link: [https://github.com/SAPRIN-MRC/ExcessMortality]. Additional materials used in the study are available upon request from the corresponding author.

## Abbreviations

AHRI: Africa Health Research Institute
BMI: Body mass index
CI: Confidence interval
COVID-19: Coronavirus disease 2019
HDSS: Health and demographic surveillance system
HIV: Human immunodeficiency virus
NCD: Non-communicable disease
PCA: Principal component analysis
PWH: People with HIV
RR: Relative rate
SES: Socioeconomic status
TB: Tuberculosis
